# A Sleeping Beauty DNA transposon-based genetic sensor for functional screening of vitamin D3 analogues

**DOI:** 10.1186/1472-6750-11-33

**Published:** 2011-04-07

**Authors:** Nicklas H Staunstrup, Nynne Sharma, Rasmus O Bak, Lars Svensson, Thomas K Petersen, Lene Aarenstrup, Karsten Kristiansen, Lars Bolund, Jacob Giehm Mikkelsen

**Affiliations:** 1 Department of Human Genetics, University of Aarhus, DK-8000 Aarhus C, Denmark; 2 Department of Disease Pharmacology, LEO Pharma, DK-2750 Ballerup, Denmark; 3 Department of Biochemistry and Molecular Biology, University of Southern Denmark, DK-5230 Odense M, Denmark; 4 Department of Biology, University of Copenhagen, DK-2200 Copenhagen N, Denmark

## Abstract

**Background:**

Analogues of vitamin D3 are extensively used in the treatment of various illnesses, such as osteoporosis, inflammatory skin diseases, and cancer. Functional testing of new vitamin D3 analogues and formulations for improved systemic and topical administration is supported by sensitive screening methods that allow a comparative evaluation of drug properties. As a new tool in functional screening of vitamin D3 analogues, we describe a genomically integratable sensor for sensitive drug detection. This system facilitates assessment of the pharmacokinetic and pharmadynamic properties of vitamin D3 analogues. The tri-cistronic genetic sensor encodes a drug-sensoring protein, a reporter protein expressed from an activated sensor-responsive promoter, and a resistance marker.

**Results:**

The three expression cassettes, inserted in a head-to-tail orientation in a Sleeping Beauty DNA transposon vector, are efficiently inserted as a single genetic entity into the genome of cells of interest in a reaction catalyzed by the hyperactive SB100X transposase. The applicability of the sensor for screening purposes is demonstrated by the functional comparison of potent synthetic analogues of vitamin D3 designed for the treatment of psoriasis and cancer. In clones of human keratinocytes carrying from a single to numerous insertions of the vitamin D3 sensor, a sensitive sensor read-out is detected upon exposure to even low concentrations of vitamin D3 analogues. In comparative studies, the sensor unveils superior potency of new candidate drugs in comparison with analogues that are currently in clinical use.

**Conclusions:**

Our findings demonstrate the use of the genetic sensor as a tool in first-line evaluation of new vitamin D3 analogues and pave the way for new types of drug delivery studies in sensor-transgenic animals.

## Background

The active form of vitamin D3, 1α,25-dihydroxyvitamin D3 (also known as calcitriol), belongs to the family of steroid hormones and has long been known for its pivotal role in calcium and phosphate homeostasis, bone metabolism, and regulation of cell growth in skin [1]. However, accumulating evidence suggests a much broader range of biological actions of calcitriol including differentiation, proliferation, and apoptosis of a wide range of cell types [2,3]. Hence, vitamin D3 deficiency has been associated with cancer development, loss of renal function, cardiovascular disease, and deregulation of immune function, supporting the role of vitamin D3 derivatives as potent antiproliferative and therapeutic agents [4,5].

**Figure 1:**
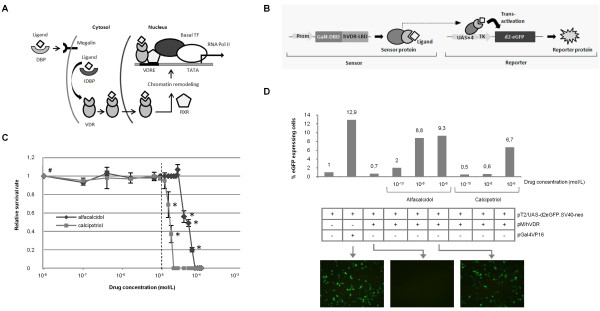
**A genetic sensor system for detection of vitamin D analogues**. **(A) **Mechanism of transcriptional activation mediated by vitamin D3. DBP, vitamin D binding protein; IDBP, intracellular vitamin D binding proteins (IDBPs); VDR, vitamin D receptor (VDR); RXR, retinoid X receptor; VDRE, vitamin D response element. **(B) **Schematic representation of the components of the sensor and mode of action. Promoters are presented as light grey arrows. **(C) **Effect of calcipotriol and alfacalcidol on cell survival at different concentrations. HaCaT cells were supplemented drug at various concentrations, demonstrating reduced *in vitro *survival at concentrations ≥10^-5 ^M. Surviving colonies were stained and counted after 8 days. The data are shown as mean values ± standard deviations. A square (#) indicates the level of survival of untreated cells, whereas stars (*) indicate evident change in morphology. The broken line indicates the maximum concentration employed in subsequent experiments. **(D) **Receptor and sensor function under transient transfection conditions. HEK293 cells were co-transfected with 1.5 μg pT2/UAS-d2eGFP.SV40-neo together with 0.5 μg pGal4VP16 or pMhVDR. The cells were left untreated or exposed to DMSO-dissolved alfacalcidol or calcipotriol at different concentrations (as indicated below the x-axis) 24 hours after transfection. After an additional 48 hours the cells were analyzed by flow cytometry. Expression of d2eGFP in untreated cells was set to 1 (left column). Representative fluorescence microscopy pictures (at 16X magnification; 48 hours after drug supplementation) of relevant combinations of transfected plasmids and drug treatment (as indicated in the table) are shown at the bottom.

Calcitriol exerts its role by binding to the ubiquitous nuclear vitamin D receptor (VDR) (see figure 1A for schematic overview). VDR, a trans-acting transcription factor belonging to the superfamily of nuclear hormone receptors [6,7], is constituted of five functional domains including an N-terminal DNA binding domain (DBD), a C-terminal ligand binding domain (LBD) and a transcription factor interaction interface. Circulating vitamin D3 is bound by vitamin D binding protein (DBP). Transport across the cellular membrane is mediated by diffusion or megalin-mediated endocytosis prior to binding of the ligand by intracellular vitamin D binding proteins (IDBPs) within the cytoplasm. Upon ligand binding, VDR undergoes a conformational change that allows interaction with other cellular proteins. The VDR-ligand complex is transported across the nuclear membrane and then forms a nuclear heterodimeric complex with the retinoid X receptor (RXR). The ligand-bound VDR-RXR complex recruits coactivators that may induce chromatin remodeling by inducing histone acetylation. VDR-RXR recognizes genomic VDR target sequences known as vitamin D response elements (VDREs) [8] and activates transcription by recruiting basal transcription factors, such as TFIIB, TATA-binding protein (TBP) and transcription-associated factors (TAFs), and RNA polymerase II [9]. VDRE motifs are found in large subset of genes that are potentially regulated by the receptor complex. Ligand-activated VDR predominately acts as a transcription inducer, but is also implicated in repression pathways by masking promoter sequences through binding of VDRE sites. Growth-promoting genes, like c-myc, may be repressed by such mechanisms [10], whereas certain interleukins, including IL-8 and IL-12, may be amenable to this type of regulation through repression of NF-kappaB-directed transcriptional activation [11-13].

The antiproliferative actions of calcitriol have prompted the development of calcitriol and synthetic vitamin D3 analogues as new drugs for treatment for various disorders including secondary hyperparathyroidism in chronic kidney disease, various cancers, osteoporosis, and hyperproliferative conditions in the skin (reviewed in [14]). Vitamin D therapy is well-established in autoimmune skin diseases, and analogues of Vitamin D3 currently represent the most frequently prescribed drugs for topical treatment of mild and moderate inflammation of the skin. The pathogenesis of psoriasis, a common chronic inflammatory skin disease, is inherently complex and characterized by alterations of various cell types. Activated skin-homing lymphocytes trigger release of cytokines and growth factors, leading to a massive inflammation response, angiogenesis and vascular dilation as well as keratinocyte hyperproliferation leading to hyperkeratosis [15-19]

Calcitriol, or synthetic derivatives, control skin homeostasis by regulating keratinocyte proliferation and differentiation [20-22] through antiproliferative mechanisms that include a variety of growth-regulating pathways, including Erk/MAPK and phosphoinositide 3-kinase/AKT signaling, that in turn suppress expression of DNA replication genes [23]. Calcitriol-induced repression of keratinocyte growth is accompanied by expression of differentiation markers such as transglutaminase and involucrin and formation of cornified envelopes [24,25]. In addition, topical application of vitamin D3 analogues may induce apoptosis of psoriatic keratinocytes [26]. The immunemodulatory actions of vitamin D3 derivatives may include transcriptional repression of cytokine production [11-13] and regulation of antigen-presenting cell functions and the capacity to modulate the T-cell repertoire and T-helper cell responses in skin [27].

The broad therapeutic potential of vitamin D3 compounds has triggered a search for new engineered compounds with improved specificity and high VDR affinity and which do not cause side-effects such as hypercalcemia, soft tissue calcification and bone resorption upon systemic administration [28]. Although topically applied vitamin D3 analogues are typically well tolerated and adverse effects seem confined to mild irritant dermatitis and only in rare cases hypercalcemia [29,30], not all psoriasis-affected individuals respond to the treatment. Disparities between vitamin D3 analogues and their biological properties may reflect specific VDR binding properties and small differences in the conformational changes of ligand-bound VDR. In different cell types, such small variations may potentially influence the interaction between the VDR-RXR complex with co-activators and co-repressors [31], ultimately causing differences in patterns of transcriptional modulation.

The efficacy of vitamin D3 analogues is increased with optimized cellular uptake, improved drug stability, high VDR affinity, and induction of ideal conformational changes in VDR. With the purpose of screening new drugs with respect to such parameters, we present here a novel technology for genetically based sensoring and functional screening of vitamin D3 analogues. By use of a genomically integratable bipartite sensor that contains both sensor and reporter modules expressed from a single genetic entity, we can monitor cellular administration and function in any cell type of interest. This type of sensor allows direct comparison of engineered vitamin D3 compounds in different cellular contexts and establishes a platform for studies of drug delivery *in vivo *by constructing transgenic animals with the sensor system.

The genetic sensor combines the well-characterized Gal4-UAS transcriptional system discovered in *Saccharomyces cerevisiae *[32] and the effective Sleeping Beauty (SB) Tc1-like transposable element reconstructed from teleost fish [33]. SB is a 'cut-and-paste' transposable element that has been developed as a gene-inserting vector for gene therapy and transgenesis [34]. As a major advantage, SB can accommodate various individual expression cassettes that are, in the context of the transposon vector, inserted into the genome in a reaction catalyzed by the SB transposase. Here, we describe the efficient mobilization and high sensitivity of a tri-cistronic SB-derived sensor expressing (i) a sensor protein, driven by a keratinocyte-specific promoter, consisting of the ligand binding domain (LBD) of the vitamin D receptor (VDR) fused to the Gal4 DNA-binding domain (DBD), (ii) a destabilized eGFP reporter protein (d2eGFP) driven by a Gal4-responsive promoter, and (iii) a selection marker gene, neo, driven by the simian virus 40 (SV40) promoter. In DNA transposon-containing keratinocytes, the integrated sensor exhibits a sensitive *in vitro *response to vitamin D3 analogues and facilitates a direct functional comparison of a set of synthetic analogues, some of which are currently in clinical use. This approach allows comparative studies of drug binding affinity, activity and delivery and may serve as a first-line tool for evaluation of new vitamin D3 analogues and as a transgenic marker for drug delivery in sensor-transgenic animals.

## Results

### Genetic sensoring of vitamin D analogues alfacalcidol and calcipotriol

To develop a sensoring system for sensitive detection of vitamin D3 analogues, we first established separate sensor and reporter vectors for drug detection by transient expression in transfected cell lines. In the sensor construct, a fusion protein containing the ligand binding domain (LBD) of the human vitamin D receptor (hVDR) fused to the Gal4 DNA-binding domain (DBD) was expressed from an SV40 promoter, whereas the reporter plasmid contained a reporter gene expressing the destabilized eGFP protein (d2eGFP) from a Gal4-responsive promoter (UAS-TK) (figure 1B). The sensor design was based on previous findings suggesting that the hVDR, upon binding by the ligand, forms heterodimers with RXR protein and via the DBD domain binds to the Gal4-responsive element UAS [35]. Here, transcription factors are recruited due to the ligand-induced conformational change of hVDR, thus leading to transcription of the d2eGFP gene (figure 1B). Given the inherent rapid turnover of d2eGFP (half life of approximately 2 hours), the signal intensity and sustainability is suspected to depend on ligand dose, stability and binding kinetics.

Calcipotriol and alfacalcidol are synthetic vitamin D3 derivates with modifications in the side chain. Calcipotriol is an approved psoriasis-treating drug marketed under the trade name Dovonex^®^, whereas alfacalcidol, marketed under the trade name One-Alpha^®^, is used in the management of vitamin D deficiencies such as osteomalacia [36,37]. These well characterized compounds were therefore utilized in the initial tests of the sensor. Using the HaCaT keratinocyte cell line as a model system, we first wanted to define an *in vitro *working range for the two compounds. A kill-curve was constructed based on a relative survival rate of naive HaCaT cells, cultured in the presence of either calcipotriol or alfacalcidol in concentrations ranging from 10^-7 ^M to 10^-4 ^M, compared to a no drug control (figure 1C). The cells were indifferent to concentrations below 10^-5 ^M for calcipotriol and below 2.5 × 10^-5 ^M for alfacalcidol above which a marked change in morphology (data not shown) and a dramatic increase in apoptosis was evident. Thus, for the succeeding studies of drug detection a maximum concentration of 10^-5 ^M was used which is comparable to *in vitro *experimental concentrations used by others [38,39].

Next, HEK-293 cells were transfected transiently with the two plasmids encoding sensor and reporter proteins, respectively, and treated with calcipotriol or alfacalcidol. As a positive control, we also co-transfected cells with the d2eGFP reporter construct together with a plasmid expressing a constitutively active transcriptional activator consisting of Gal4-DBD fused with the herpes simplex virus-derived virus protein 16 (VP16). Transfected cells were analyzed by flow cytometry two days after transfection. As shown in figure 1D, d2eGFP expression was markedly increased by the presence of the Gal4VP16 activator, demonstrating VP16-directed induction of transcription from the UAS-TK promoter. Also as expected, cells transfected only with the d2eGFP reporter plasmid were in general negative for expression of the reporter protein, although a vague background of d2eGFP expression could be detected (data not shown) indicative of limited leakiness of the UAS-TK promoter. With increasing concentrations of calcipotriol and alfacalcidol, expression of d2eGFP was induced in cells co-transfected with both sensor and reporter plasmids. For calcipotriol, the reporter was activated at the highest analyzed drug concentration (10^-6 ^M). Alfacalcidol activated the sensor system most potently, and robust d2eGFP induction could be monitored with a drug concentration of 10^-8 ^M and even 10^-10 ^M (figure 1D). In summary, these findings provided evidence of the detection of a cellular uptake of vitamin D3 analogues by a split two-component genetic sensor system.

### Generation of a vitamin D sensor-reporter platform in a SB DNA transposon vector

**Figure 2:**
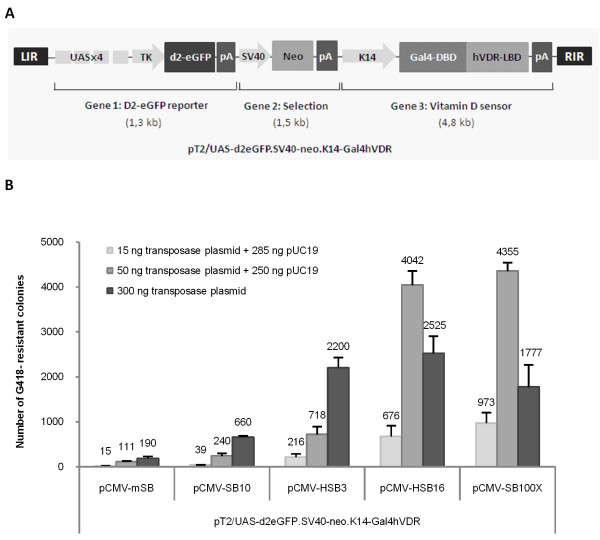
**Effective transposition of the tri-cistronic receptor-sensor SB DNA transposon in HaCaT cells**. **(A) **Schematic representation of the SB transposon-based sensor. Modules of the sensor are flanked by the left and right inverted repeat (LIR and RIR, respectively) of the SB transposon. The 17-bp Upstream Activating Sequence quarto-repeat (UASx4) assists the minimal thymidine kinase (TK) promoter in expressing the destabilized enhanced green fluorescence (d2eGFP) protein flanked by a Simian virus 40 (SV40) polyadenylation sequence. The selection cassette consists of a SV40 promoter driving transcription of the neomycin resistance gene (Neo) tailed by a SV40 polyadenylation sequence. The receptor component consists of the skin-specific keratin 14 (K14) promoter driving expression of the chimeric Gal4 human vitamin D receptor (Gal4hVDR) gene flanked downstream by the K14 polyadenylation sequence. The K14 promoter restricts expression to keratinocytes and derived cell lines or the stratum basale of the skin. **(B) **Genomic insertion of the genetic sensor catalyzed by a panel of transposases. The transposition assay was carried out with various transposases at different concentrations. HaCaT cells were transfected with 1.7 μg pT2/UAS-d2eGFP.SV40-neo.K14-Gal4hVDR and 300 ng, 50 ng or 15 ng plasmid encoding mSB, SB10, HSB3, HSB16 or SB100X. Additionally, 250 ng or 285 ng pUC19 was included as stuffer to ensure that equal amounts of DNA were used in each transfection. Transposition activity was measured by counting the number of G418-resistant colonies 14 days post-transfection. All experiments were performed in triplicates and are the data shown here as mean values ± standard deviations.

The SB DNA transposon system facilitates efficient host genome integration of genetic cargo with a total size of more than 10 kb. The unique properties of SB as a 'cut-and-paste' DNA transposon allow transposase-directed co-mobilization of a set of separate gene cassettes, each carrying their own promoter, contained in the context of a single transposon vector. As a result, the gene cassettes are inserted side-by-side in a single chromosomal locus. Conventional integrating viral vectors may not as easily be modified to contain three gene cassettes since viral transduction may be inhibited by the presence of several promoters and the length of the construct. To engineer an integratable genetic sensor, we constructed a tri-cistronic SB vector (designated pT2/UAS-d2eGFP.SV40-neo.K14-Gal4hVDR) accommodating (i) the UAS-d2eGFP reporter driven by the UAS-TK promoter, (ii) a neomycin resistance gene expressed from an SV40 promoter, and (iii) the Gal4hVDR sensor expressed from a keratin 14 (K14) promoter (figure 2A). Use of the K14 promoter would confine expression of the sensor protein to cell types with a keratinocyte origin such as the human keratinocyte cell line HaCaT (K14 activity verified by RT-qPCR, data not shown), or *in vivo *to the stratum basale of the epidermis.

The transposition efficiency of the tri-cistronic SB vector was evaluated by transfecting HaCaT cells with 1.7 μg pT2/UAS-d2eGFP.SV40-neo.K14-Gal4hVDR together with 15, 50 or 300 ng of transposase-encoding plasmid. A panel of five transposase plasmids (pCMV-mSB, pCMV-SB10, pCMV-HSB3, pCMV-HSB16, and pCMV-SB100X) was utilized encoding transposases of varying functional potencies ranging from the inactive mutated mSB to the hyperactive SB100X variant. The different transposases were expressed from otherwise identical plasmids by the CMV promoter.

Numbers of G418-resistant colonies (figure 2B) indicated that the neo expression cassette was inserted markedly more frequently in the presence of active transposases relative to the inactive mSB, indicating that genomic integration of the tri-cistronic vector was efficiently accomplished by a transposase-directed mechanism. Using a medium dose (50 ng) of transposase-encoding plasmid the relative activity of the five transposase variants was as follows: mSB < SB10 < HSB3 < HSB16 < SB100X. In contrast, in the higher-dose experiment (300 ng plasmid) the presence of HSB16 resulted in the highest number of colonies. This finding was a likely indication of an inhibitory effect of increased concentrations of the transposase (a phenomenon referred to as 'overproduction inhibition' [40]) when SB vector insertion was catalyzed by SB100X. At a low transposase dosage (15 ng plasmid) the transposition efficiency was generally low, although the difference between mSB and SB100X was most pronounced at this dosage (65-fold difference, p = 0.0043). Also, the concentration of mSB seemed to influence the rate of random integration. Provided the widely accepted fact that mSB is catalytically inactive, this finding suggested that mSB to a limited degree supported random plasmid insertion.

### Detection of Vitamin D3 analogues by integrated SB sensor

**Figure 3:**
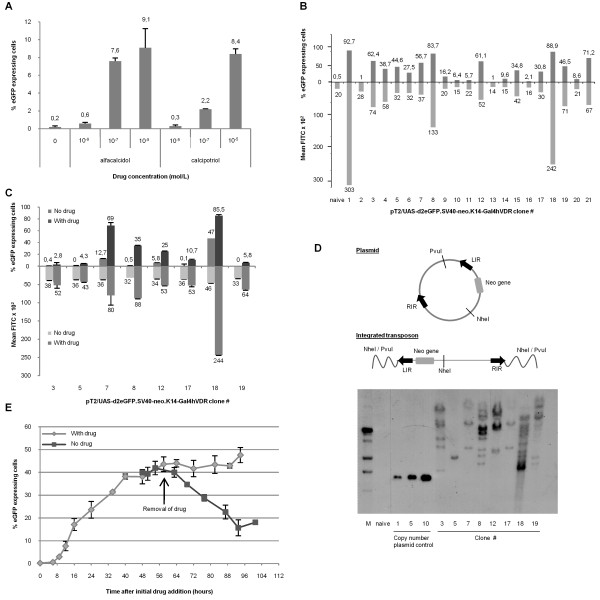
**Generation of stable and highly inducible sensor clones with rapid increase and decline of signal**. **(A) **Sensor induction in HaCaT keratinocytes. HaCaT cells were co-transfected with 1.5 μg pT2/UAS-d2eGFP.SV40-neo.K14-Gal4hVDR and 0.5 μg pCMV-SB100X. Sensor-containing cells were exposed to DMSO-dissolved vitamin D3 analogues at different concentrations for 48 hours and analyzed by flow cytometry. **(B) **Induction of sensor in individual HaCaT clones. Cells were exposed to DMSO-dissolved 10^-5 ^M alfacalcidol and analyzed by flow cytometry after 2 days. D2eGFP expression levels are shown in the first quadrant and mean FITC intensity in the second quadrant. Registered signal in the FITC channel from un-transfected (naive) HaCaT cells served as reference. **(C) **Sensor activity in the absence and presence of drug. Eight clones selected among the 21 sensor-containing clones were incubated 48 hours in absence or presence of 10^-5 ^M alfacalcidol and subsequently evaluated by flow cytometry. **(D) **Southern blotting shows variable integration patterns among the vitamin D3-responsive clones. **(E) **Kinetics of the sensor upon exposure to vitamin D3 analogues. Cells from clone #8 were initially split in two sets that were grown in parallel. With regular time intervals medium containing 10^-5 ^M alfacalcidol was added to the cells. At 52 hours incubation one set was relieved from the compound and cultured in drug-free media. At the end of the experimental time frame, the cells were analyzed by flow cytometry. The experiments in (A), (B), and (E) were conducted in triplicate, and data are shown as mean values ± standard deviations.

To evaluate the sensor capacity of the integrated tri-cistronic sensor-reporter system, we first tested a population of pooled G418-resistant HaCaT clones stably transfected with pT2/UAS-d2eGFP.SV40-neo.K14-Gal4hVDR for responsiveness to calcipotriol and alfacalcidol (figure 3A). Exposure to the drug resulted in a significant increase in fluorescence in a concentration-dependent manner. Thus, at 10^-5 ^M alfacalcidol the percentage of d2eGFP-positive cells was roughly 43 times higher than without the drug (p ≤ 0.0001), about 38 times higher than at a concentration of 10^-7 ^M (p ≤ 0.0001) and roughly 3 times higher than at a concentration of 10^-9 ^M (p = 0.0001). A similar pattern was evident for calcipotriol, although alfacalcidol was more potent in the assay.

We next isolated and expanded individual clones containing the SB sensor inserted by the SB100X transposase. Twenty-one clones were grown in alfacalcidol-containing medium (10^-5 ^M) and subsequently analyzed by flow cytometry (figure 3B). The collection of clones displayed a highly heterogeneous response to alfacalcidol. Hence, the amount of d2eGFP-positive cells ranged from background level to 92.7% of the analyzed cells. This clonal difference was paralleled by substantial differences in mean fluorescence (figure 3B). This variation in responsiveness among individual clones offered an explanation for the relatively low d2eGFP induction level that was observed with pooled clones (figure 3A). Based on their responsiveness to alfacalcidol, 10 clones were selected for further flow cytometric analyses. Of these 10 clones, clones #1 and #4 were impaired in growth and survival and were discontinued. The remaining 8 clones were examined in the absence or presence of 10^-5 ^M alfacalcidol (figure 3C). For all clones, expression of d2eGFP was induced with statistical significance by the presence of alfacalcidol relative to the no drug control. However, the un-induced level of d2eGFP varied considerably between clones, demonstrating some leakiness that most probably was dependent on vector copy number and the context of the insertion sites in each individual clone.

Southern blot analysis of genomic DNA isolated from the 8 clones unveiled SB vector copy numbers ranging from 1 to approximately 10 copies per clone (figure 3D). For some clones there was coherence between the expression profile and copy number. Clones #5 and #18 for example were characterized by low and high sensor activity, respectively, corresponding to the detection of 1 and numerous insertions in the two clones, respectively. In other clones, high d2eGFP expression was achieved with few copies of the vector (e.g. clone #7 which had only 4 insertions), whereas low expression could be detected in clones with high copy numbers (e.g. clone #19). This suggested that the receptor-sensor system was sensitive to the properties of the insertion site perhaps due to varied accessibility to the vector in non-permissive heterochromatin and permissive euchromatin. Interestingly, the single integration observed for clone #5 gave rise to a significant increase in d2eGFP expression upon alfacalcidol induction (p = 0.0007). This finding demonstrated that the system can work in *cis *where a ligand-activated sensor protein expressed from one cassette in the SB vector can induce expression from another cassette in the same vector, adding to the notion of a highly sensitive system.

To get a better understanding of the system kinetics, we periodically monitored d2eGFP expression in clone #8 grown in the presence of 10^-5 ^M alfacalcidol (figure 3E). A measureable level was obtained already after 6 hours after the initial exposure to alfacalcidol followed by a nearly linear increase in expression before reaching a plateau about 60 hours after the initial induction. In the presence of alfacalcidol the d2eGFP level remained unchanged for the duration of the experiment (almost 4 days). However, removal of drug, resulting in an expected gradual depletion of intracellular drug, caused a simultaneous rapid decline of eGFP expression. Hence, 39 hours after drug removal the d2eGFP level had decreased by 63% (p = 0.0013). In conclusion, such rapid onset and offset of d2eGFP expression allow expeditious drug evaluation and sensitive sensoring of vitamin D analogues.

### Functional screening and comparison of vitamin D3 analogues by cell lines containing the SB-based genetic drug sensor

**Figure 4:**
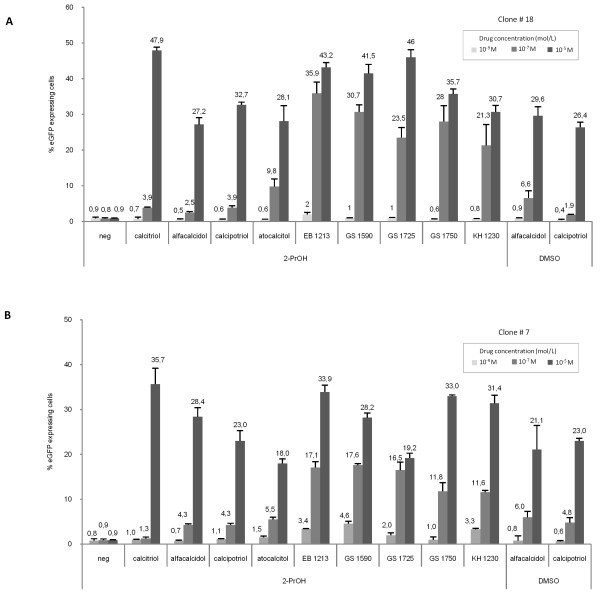
**Sensor-based screening of 10 vitamin D3 analogues revealed compounds more potent than therapeutically approved drugs**. **(A) **Efficacious sensor induction in clone #18. Cells derived from Clone #18 were exposed to vitamin D3 analogues at concentrations 10^-9 ^M, 10^-7 ^M and 10^-5 ^M. As indicated below the chart, the vitamin D3 analogues had been dissolved in either isopropanol (2-PrOH) or DMSO. After 48 hours the percentage of d2eGFP positive cells was measured by flow cytometry. Untreated (neg) cells served as control. A clear dose-dependent outcome is visible for all drugs. The experiment was conducted in triplicate, and data are indicated as mean values ± standard deviations. **(B) **Sensor induction in clone #7. Cells from clone #7 were treated as described for clone #18. The overall tendency is highly similar for clone #7 and #18.

To assess the potency of various vitamin D3 analogues, some of which have not to date been approved for therapeutic use, clones #7 and #18 were chosen for additional experimentation. The clones were grown in the presence of vitamin D3 analogues (diluted in 2-PrOH) in concentrations ranging from 10^-9 ^M to 10^-5 ^M. The cells were analyzed by flow cytometry after two days of treatment with the agonist (figure 4). The un-induced clone #18 (grown in the absence of drug) provided the baseline d2eGFP level. Furthermore, all settings and gates were identical for all measurements. For both cell lines, all analyzed vitamin D3 analogues resulted in induced d2eGFP expression although the response in clone #18 in general was stronger than the response in clone #7 (compare figures 4A and 4B). This finding matched the previous observations for the two clones (figure 3B and 3C). At the lowest applied concentration (10^-9 ^M) EB1213, GS1590, and KH1230 showed a statistically significant elevated d2eGFP level in clone #7 (p = 0,001, p = 0,001, p = 0,038, respectively) with a similar tendency in clone #18. Thus, at this low concentration as many as three analogues out-performed the clinically used calcitriol and calcipotriol in terms of reactivity and sensor induction. At 10^-7 ^M, the difference in potency between the established compounds, like alfacalcidol and calcipotriol, and the remaining vitamin D3 variants became even more profound. Under these conditions, the analogues EB1213 through KH1230 (as presented in figure 4) resulted in a marked increase in d2eGFP expression levels. Interestingly, EB1213 reached a level of expression that was 9 times higher than the level measured with calcipotriol (p = 0.0001) in clone #18. At the highest concentration (10^-5 ^M) the differences in sensor induction between the compounds was less clear and, notably, calcitriol displayed the greatest effect under these conditions.

A hallmark of vitamin D3 analogues in psoriasis amelioration is the ability to inhibit proliferation of keratinocytes. To correlate sensor inducibility with their capacity to inhibit cellular proliferation, we measured the half inhibitory concentration (IC_50_) for each of the compounds on HaCaT proliferation (Table 1). Interestingly, we found a correlation between the magnitude of the IC_50 _value and the inducibility of the genomically inserted SB-derived sensor. Hence, these findings suggest that this novel genetic sensor provides an efficient method of screening new drugs and a quick clear indication of the therapeutic potency of vitamin D3 analogues at the cellular level.

**Table 1:** Coherence between sensor inducibility and cellular proliferation inhibition by the vitamin D3 analogues

	Vitamin D3 compound								
	
	Calcitriol	Alfacalcidol	Atocalcitol	Calcipotriol	GS 1750	GS 1590	GS 1725	KH 1230	EB 1213
**Relative IC50**^**-1 (1)**^	1.0	1.1	1.6	2.9	2.9	32.3	35.7	76.9	250

**Relative potency**^**(2) **^**Clone #18**	1.0	0.6	2.7	1.0	7.2	7.9	6.0	5.5	9.2

**Relative potency**^**(2) **^**Clone #7**	1.0	3.3	4.2	3.3	9.1	13.5	12.7	8.9	13.2

^(1) ^The half maximal inhibitory concentration (IC_50_) is a measure of the capability of the compounds to inhibit proliferation of HaCaT cells. For a direct comparison, the inverse value (IC_50_^-1^) is depicted here normalized to the response obtained during calcitriol exposure. IC_50 _values were measured for drug concentrations ranging from 10^-11 ^to 10^-7 ^M.

^(2) ^The relative capacity of each compound to induce d2eGFP expression from the sensor is shown for each of the two clones #7 and #18 (data derived from figure 4). Induction of d2eGFP was measured at a drug concentration of 10^-7 ^M.

## Discussion

Functional testing of new vitamin D3 analogues and formulations for improved systemic and topical administration depends on sensitive screening methods that allow evaluation of cellular uptake, functional intracellular properties and, in case of topical administration, drug delivery to the skin. In this study, we describe a new approach for genetically sensoring the activity of vitamin D3 analogues in keratinocytes or in any cell type of interest. We establish an integratable reporter system, allowing rapid assessment and comparison of the potency of vitamin D3 analogues, some of which are currently in clinical use in treatment of psoriasis. The genetic sensor features two functional components, a vitamin D sensor protein consisting of the vitamin D receptor fused to the DNA-binding domain of Gal4 (expressed from a keratinocyte-specific K14 promoter) and a reporter module from which d2eGFP is expressed under control of an inducible promoter containing four copies of the UAS-element. This screening technology takes advantage of the genome-integrating capacity of the SB DNA transposon which facilitates accurate and near-random genomic insertion of genetic cargo of interest [40]. By mobilizing this tri-cistronic transposon-based vector in cells of interest, it is possible to incorporate all elements of the genetic sensor, including the sensor and reporter components and a selective marker gene, in a single genomic locus, allowing easy incorporation of sensor functions in any cell type of interest.

Based on our interest in improving psoriasis treatment and methods for analyzing the functional properties of vitamin D3 analogues in keratinocytes, we generated HaCaT keratinocyte cell lines with the genetic sensor stably integrated by transposition mediated by the hyperactive SB100X transposase [41,42]. Despite the fact that the tri-cistronic transposon vector is fairly long (8.3-kb) and, moreover, that HaCaT cells are difficult to transfect, robust transposition of the genetic sensor was easily achieved allowing production of stably transfected cell lines. By utilizing the SB system, instead of relying on random plasmid breakage and insertion by recombination, it was ensured that the expression cassettes were undamaged during the insertion process. Among a panel of transposase variants, the hyperactive SB100X transposase was the most efficient. However, for this particular transposase, we also observed an inhibitory effect of increasing the amount of transpose-expressing plasmid, indicating that transposition catalyzed by this transposase in cultured keratinocytes is regulated by overproduction inhibition. Such inhibition has previously been observed for all SB transposase variants [43], but appear in hard-to-transfect cells to be evident only for the most active transposases under the given conditions.

Drug-resistant clones harboring the tri-cistronic transposon vector were isolated and analyzed for the sensitivity of sensoring vitamin D derivatives. Among a group of 21 clones analyzed for induction of d2eGFP expression, only 3 clones did not express the marker gene in the presence of a vitamin D3 analogue. The remaining clones responded after supplementation of analogues but proved highly heterogeneous concerning the ability to induce d2eGFP expression. To some degree, this feature may be attributed to variations in copy-number of the tri-cistronic transposon vector (ranging from 1 to approximately 10 copies) but may also reflect genomic differences affecting gene expression from the sensor. Importantly, we identified a single clone (#5) with a single copy of the genetic sensor. Successful Vitamin D-directed induction of d2eGFP expression in this clone demonstrated that the genetic sensor was able to work *in cis *and that one sensor module was sufficient to obtain a detectable readout.

A dose-dependent response to the addition of alfacalcidol and calcipotriol could be recorded within a range of drug concentrations used in previous functional studies of vitamin D3 analogues [6,44,45]. Other Gal4-receptor hybrids also exhibited dose-responsiveness [46], emphasizing the dynamic working range of the Gal4-UAS system and its suitability for drug comparative studies. The dose-response assay showed evidence of a rapid onset of d2eGFP signal upon cell exposure to vitamin D3 analogues, which is in the interest of a sensitive and rapid drug screening methodology. More importantly, information regarding the exact time of onset, slope and peak intensity induced by exposure to the drug allows comparative analyses of functional properties related to the kinetic nature of a given drug and its cellular permeability. By tracking the decline in d2eGFP expression after drug removal one may provide insight into the stability of the VDR-RXR-UAS complex and bioavailability of the drug. Such properties may give comparative indications of drug clearance by metabolism or sequestering by endogenous proteins.

Genetic sensoring represents a new approach for functional screening of synthetic derivatives of vitamin D3. To demonstrate such potential, we screened eight different vitamin D3 analogues, some of which are currently used in psoriasis treatment and some of which are more recently developed drugs. By using two highly responsive HaCaT cell line clones (#7 and 18) harboring four or more copies of the genetic sensor, we identified a group of compounds which activated the sensor very potently. Notably, by use of the two clones quite similar activation patterns were seen for each of the tested compounds. Several of the tested compounds were found to activate the sensor with higher efficiency than current drugs like calcitriol and calcipotriol. This difference was evident at the lower drug concentrations, supporting the notion that these drugs may efficiently bind and promote a beneficial conformational change of the VDR in keratinocytes. Such increased activation of the sensor was paralleled by an increased capacity of these particular drugs to inhibit proliferation in HaCaT cells, as demonstrated by increased IC_50_^-1 ^values for all these drugs. It is important to note that the growth inhibitory effect for keratinocytes *in vitro *does not prevail until 4-6 days after addition of the drug and therefore is not expected to influence the potency estimation [44]. However, high potency to activate the vitamin D receptor or inhibiting keratinocyte proliferation, are only some parameters to consider before selecting a novel vitamin D3 analogue for further drug development.

The specific properties that enable a given vitamin D3 analogue to perform superior to calcitriol may fall into three categories (i) alterations in the pharmacodynamics including increased affinity for VDR, prolonged stability of the receptor-DNA complex, a changed receptor conformation or epigenetic changes, (ii) pharmacokinetic influences like improved half-life through a slower metabolic clearance, diminished absorption by cellular factors such as serum vitamin D binding protein (DBP) resulting in higher bioavailability, and improved cellular access, or (iii) pharmacogenetic aspects including transcriptional changes and alterations in the cellular environment. In addition, secondary effects that are not mediated by the transcription-factor activity of VDR may also be involved. Hence, e.g. the Raf1-MAPK pathway which plays a pivotal role in the mechanism of keratinocyte growth is stimulated by calcitriol. Thus, before selecting a novel vitamin D3 analogue for drug development, several different aspects and properties of a compound must be considered. The genetic sensor for *in vitro *assessment of VDR activation may address some of these parameters as rapid functional selection among a large library of new potential drugs. Vitamin D derivatives with high sensor activation capacity may subsequently be further scrutinized in more elaborate experimental settings including keratinocyte inhibitory studies and protein interaction studies. However, to verify if such findings *in vitro *have any relevance, a more subtle *in vivo *assessment may be necessary, for example in preclinical studies using appropriate animal models [47]. One such model is the xenograft transplantation model in which human psoriatic skin is grafted onto immunodeficient mice, allowing assessments of various systemic and topical treatments [48,49].

Implementation of the sensor technology in animal studies may provide new opportunities for combined studies of VDR modulation and drug kinetics and delivery. Numerous transgenic animals have been generated by use of the SB transposon system, [41,50,51], making it reasonable to envision production of sensor-transgenic animals. Recently, we demonstrated the use of SB transposon vectors for transgenesis of cloned Göttingen minipigs [52], paving the way for introduction of SB-based genetic sensors in the pig. Given the fact that the skin of pigs has many anatomical and physiological similarities with human skin, the establishment of an animal model for genetically sensored vitamin D delivery and permeability in skin may have a strong potential in studies of drug delivery and novel drug formulations with relevance for topical treatment in humans. With the rapidly emerging applications of vitamin D3 analogues in treatment of human diseases, including cardiovascular diseases, inflammatory disorders, and cancer, the interest in a sensitive *in vivo *screening approach should not be restricted to topical drug administration and psoriasis. Notably also, the VDR component of the system that we describe herein is easily interchangeable with other receptors of interest, making the concept of an integratable sensor approach highly versatile.

## Conclusions

We demonstrate the use of a DNA transposon-based genetic sensor system, allowing for rapid functional screening of vitamin D3 analogues in an *in vitro *setting. The sensor module was easily inserted into the genome of keratinocytes, allowing comparative functional studies of vitamin D3 analogues in a keratinocyte background. HaCaT cells stably expressing the sensor and reporter components of the sensor showed potent activation of the sensor in a fashion that was dependent on the drug and its concentration, thereby facilitating assessment of drug kinetics and dynamics. A total of eight analogues were tested against the active form of vitamin D3 (calcitriol). Several of the analogues proved more potent than calcitriol and exhibited significant signal to noise ratios even at low concentrations (10^-9 ^M). Due to the in-built possibility of exchanging each component individually, the sensor system can be adapted to accommodate other receptors of interest and/or possess an alternative cell-specific expression profile, with the potential of enabling high-throughput screening of candidate drugs in a time-saving and cost-effective manner. The creation of genetically engineered animals transgenic for the genetic sensor will pave the way for *in vivo *investigations of drug potency and administration, allowing for more elaborate comparative studies of vitamin D3 analogues.

## Methods

### Generation of a tri-cistronic receptor-sensor construct

All the basic plasmids (pUAS-tk-luc (57), pd2eGFP1 (Clontech Laboratories, Inc., Mountain View, CA, USA), pM (Clontech Laboratories, Inc., Mountain View, CA, USA) and pG3Z-K14 [53]) were present in house and the pSG5-VDR plasmid was provided by courtesy of Leo Pharma, Denmark. pT2/SV40-neo has previously been described [54]. pT2/SV40-neo was initially modified to remove the restriction sites SacI (position 107) and HindIII (position 404), and PmeI and SacI sites were introduced at position 413 and 2069, respectively. The UAS-tk promoter was released from pUAS-tk-luc by PvuII/XhoI digestion and inserted into Ecl36II/SalI-digested pd2eGFP1. The resulting UAS-tk-d2eGFP was PCR-amplified and inserted in PmeI-digested pT2/SV40-neo, resulting in the construct designated pT2/UAS-d2eGFP.SV40-neo. A fragment containing hVDR was PCR-amplified from pSG5-VDR and cloned into EcoRI/XbaI-digested pM. The resulting Gal4hVDR module was released by AvrII/XbaI digestion and incorporated in XbaI-digested pG3Z-K14. The K14-Gal4hVDR fragment was isolated by SacI/HindIII digestion and inserted into SacI/HindIII-digested pT2/UAS-d2eGFP.SV40-neo resulting in the final sensor construct designated pT2/UAS-d2eGFP.SV40-neo.K14-Gal4hVDR.

### Generation of cell clones expressing the sensor components

For stable and transient transfections, HaCaT or HEK293 cells were seeded in 6-well plates at a density of 2 × 10^5^/well. The preceding day the cells were transfected using Fugene 6 (Roche, Basel, Switzerland) according to manufacturer's protocol utilizing a 6 μL:2 μg fugene:total plasmid ratio for HaCaT and 3 μL:2 μg for HEK293 cells. The following day the cells were re-seeded in P10 dishes at varying dilutions. After another 24h, the cells were subjected to G418 (Invitrogen Ltd, Paisley PA4 9RF, UK) selection (0,6 mg/mL) for 14 days. Subsequently, the resistant colonies were either stained with methylene blue (Sigma-Aldrich, St. Louis, MO, USA) for transposition efficiency assays or pooled or isolated for system evaluations.

### RT-qPCR on pooled sensor-containing clones

HaCaT cells were transfected as described above; 1.5 μg pT2/UAS-d2eGFP.SV40-neo.K14-Gal4hVDR (or pT2/SV40-neo.K14-Gal4hVDR) + 0.5 μg pCMV-SB100X, the latter expressing the SB100X transposase. RNA from G418-resistant colonies was extracted through a spin-column (RNeasy Kit, QIAGEN, Valencia CA, USA) according to manufacturer's instructions. Extracted RNA was DNase-treated (DNA-free, Ambion, Foster City, CA USA) in accordance with protocol. 100 ng DNA-free RNA was subsequently utilized in the cDNA synthesis (Bio-Rad, iScript cDNA synthesis kit, Hercules, CA, USA). Two μL of the cDNA synthesis mix was employed in the qPCR reaction together with 6 pM Gal4 primer: 5'-AGTGCTCCAAAGAAAAACCGA-3' (forward) and Gal4 primer: 5'-GGTCTTCTCGAGGAAAAATCAG-3' (reverse) or ACTB primer: 5'-CTGGCACCACACCTTCTACA (forward) and ACTB primer: 5'-GGTCATCTTCTCACGGTTGG-3' (reverse) and 10 μL mastermix (DyNAmo HS SYBR Green qPCR kit, Finnzymes, Espoo, Finland). Amplification was conducted on a Roche LightCycler 480 under the following conditions: 1 × 95°C for 5 min; 45 × 95°C for 20 sec, 55°C for 20 sec, 72°C for 30 sec; continuous melting. Ct values were calculated from the "Fit Point Method" and relative quantification was conducted by normalization to the housekeeping gene ACTB (R = 2^ΔCtsample^/2^ΔCtreference^).

### Southern blot analysis

Genomic DNA was prepared from cell pellets following NaCl extraction and ethanol precipitation. 15 μg genomic DNA was digested overnight with NheI (a single site within the transposon) and PvuI (cleaves within the bacterial backbone, resulting in a distinct band with a length of 4585 bp in case of plasmid DNA). Genomic DNA of non-transfected cells was used as a negative control. Genomic DNA of non-transfected cells spiked with plasmid DNA corresponding to 1 copy/cell (32 pg plasmid DNA), 3 copies/cell (96 pg plasmid DNA), or 10 copies/cell (320 pg plasmid DNA) was used as a positive control. The digested DNA was electrophoresed in a 0.8% agarose gel and transferred to a Hybond membrane (GE Healthcare, Buckinghamshire, UK). The membrane was hybridized overnight using a Neo-specific [α-32P] dCTP-labelled probe.

### Flow cytometry and dose-response analysis on sensor-harboring cells

Clones containing the integrated sensor system were seeded in 24-well plates at a density of 2 × 10^4 ^cells/well and maintained in DMEM 10% FCS at 37°C and 5% CO_2_. For induction assays, vitamin D3 analogues (concentrations ranging from 10^-9 ^to 10^-5 ^M) were added after 24 h and left for additional 48 h. In the dose-response assay, the vitamin D3 analogue (10^-5 ^M) was added at different time points. The drug was either left on the cells for the duration of the experiment or removed at various time points commenced after 48 h of induction. For flow cytometry, the cells were trypsinized and resuspended in 500 μL PBS (no MgCl_2_, no CaCl_2_) and added 4 μg/mL (final concentration) propidium iodide (Sigma-Aldrich, St. Louis, MO, USA). Subsequently, 10,000 events were analyzed on a BD FACSAria machine using BD FACSDiva software. In all experiments measuring drug-induced gene activation, a negative control group (naïve) was included. Cells in this group were grown in normal medium without addition of 2-PrOH or DMSO.

### Vitamin D drugs

Calcitriol [55] and vitamin D3 analogues (alfacalcidol [56], calcipotriol [57], atocalcitol, EB1213 [1,14], GS1590 [58], GS1725 [14], GS1750 (unpublished), KH1230 [59]) were either dissolved in 2-PrOH at 4 mM or DMSO at 10 mM.

### Assessment of drug-induced cell death

Non-transfected HaCaT cells were seeded in 6 well plates at 100 cells per well. After 3 days in medium with various concentrations (ranging from 10^-7 ^to 10^-4 ^M) of calcipotriol or alfacalcidol was supplied. After an additional 5 days the surviving colonies were stained with methylene blue and counted. A kill-curve was constructed with untreated cells as reference.

### Proliferation inhibitory assay

HaCaT cells were propagated in Phenol Red-free Dulbecco's modified Eagle's medium supplemented with 5% FCS, 2 mM glutamine, 100 IU/mL penicillin, and 100 pg/mL streptomycin. The cells were incubated in a humidified atmosphere at 37°C in the absence and presence of the test compounds (10^-11 ^to 10^-7 ^M). After 120 hr of incubation, DNA synthesis was determined by incorporation of ^3^H-labelled thymidine. Each sample was tested in quadruplicate, and the concentration of test compound resulting in 50% inhibition (IC_50_) of DNA synthesis was calculated from the dose-response curve.

### Statistical analysis

All p-values were calculated by a two-tailed Student's t-test to test the null hypothesis of no difference between the compared groups. The assumption of equal variances was tested by the F-test. In all statistical analyses, p-values < 0.05 were considered significant.

## Competing interests

LSV and TKP are employed by LEO Pharma A/S, Ballerup, Denmark.

## Authors' contributions

NHS conducted all experiments not listed below and drafted the manuscript, NS and ROB performed the Southern blot analysis and assisted to the experimental design, LS and TKP helped devise the experimental design and participated in finalizing the manuscript, LA and KK undertook the initial plasmid cloning steps, LB helped conceiving the study. JGM played a central role in the design and coordination of the study and wrote the manuscript together with NHS. All authors read and approved the final manuscript.
